# Physical activity has decreased in Finnish children and adolescents from 2016 to 2022

**DOI:** 10.1186/s12889-024-18854-7

**Published:** 2024-05-18

**Authors:** Pauliina Husu, Kari Tokola, Henri Vähä-Ypyä, Harri Sievänen, Sami Kokko, Jari Villberg, Tommi Vasankari

**Affiliations:** 1 The UKK Institute for Health Promotion Research, Kaupinpuistonkatu 1, Tampere, FI-33500 Finland; 2 Faculty of Sport and Health Sciences, Research Centre for Health Promotion, University of Jyväskylä, PL 35, Jyväskylä, FI-40014 Finland; 3 Faculty of Medicine and Health Technology, Tampere University, Kalevantie 4, Tampere, FI-33014 Finland

**Keywords:** Physical activity, Sedentary behavior, Accelerometry

## Abstract

**Background:**

Varying trends in children’s and adolescents’ physical activity (PA) have been reported during the last 10–20 years. Trends in sedentary behavior (SB) have been studied only rarely. The purpose of the present study was to describe population-based trends in accelerometer-measured PA, standing and SB, among Finnish 7-15-year-old children and adolescents, and to evaluate the potential influence of the COVID-19 pandemic on these behaviors.

**Method:**

A cross-sectional population-based Finnish school-aged physical activity Study (FSPA) measured daily steps, vigorous (VPA), moderate (MPA), moderate-to-vigorous (MVPA), light physical activity (LPA), standing, and SB by an accelerometer for seven consecutive days in 2016, 2018, and 2022 (*n* = 7.080, 57% girls). The data was analyzed by multivariate regression analysis.

**Results:**

In 2016, participants took on average 10.305 steps per day, and spent 0:15 (h: min) in VPA, 1:37 in MPA, 1:52 in MVPA, 3:48 in LPA, 0:55 in standing and 7:52 in SB. From 2016 to 2018, daily steps, MPA, LPA, and standing increased [229 steps (95% Confidence Interval, CI 70–387), 0:03 (CI 0:01 − 0:04), 0:11 (CI 0:09 − 0:14), and 0:07 (CI 0:05 − 0:08), respectively], while VPA and SB decreased [0:01 (CI 0:00–0:02) and 0:20 (CI 0:16 − 0:24), respectively]. From 2018 to 2022, daily PA and standing declined [751 steps (CI 562–939), VPA 0:02 (CI 0:01 − 0:03), MPA 0:09 (CI 0:07 − 0:11), MVPA 0:11 (CI 0:09 − 0:14), LPA 0:08 (CI 0:05 − 0:11), and standing 0:01 (CI 0:01 − 0:03)] while SB increased 0:21 (CI 0:16 − 0:25) indicating potential influence of the pandemic.

**Conclusions:**

Children and adolescents became physically less active from 2016 to 2022. The potential effects of the COVID-19 were seen as declined PA and increased sedentariness from 2018 to 2022.

**Supplementary Information:**

The online version contains supplementary material available at 10.1186/s12889-024-18854-7.

## Background

Regular physical activity (PA) is important for children’s and adolescents’ healthy growth and development [1], whereas long-term sedentary behavior (SB) confer deleterious effects [2, 3]. At least moderate-to-vigorous intensity PA (MVPA) shows more consistent and robust relationships with health indicators than lower intensity PA [4]. School-aged children and adolescents are recommended to do at least 60 min of MVPA daily [1], and PA can be accrued in small doses throughout the day [4].

Currently, most children and adolescents do not meet the recommendations for PA [4–6], and there are substantial country- and region-specific differences in PA across Europe [6]. A meta-analysis of longitudinal device-based studies among adolescents showed a 7.4 min decline in daily MVPA over a four-year mean follow-up, which equated to 17% of the mean baseline estimate [7]. A systematic review covering a wider time frame reported a decline in total PA between 1995 and 2017, especially among adolescents [8]. According to a systematic review and meta-regression, daily MVPA accumulating during the school days has declined on average by 15 min during 2003–2010, plateaued thereafter, and increased by approximately 5 min during 2015–2019 [9]. Two recent reviews, mainly based on self-reported PA among children and adolescents during the COVID-19 pandemic showed decreasing trends [10, 11]. Both the duration and frequency of PA have decreased, concerning especially organized sports, as structured sports programs and facilities were closed [10, 11]. These reviews included studies that were published before fall 2021 [10, 11], which restricts their ability to assess PA after the pandemic-related restrictions were dismantled.

Most previous studies focusing on changes in children’s and adolescents’ accelerometer-measured PA and SB have used MVPA as the main outcome. Light PA and SB have been studied rarely and standing has not been assessed. To our knowledge, the number of population-based studies reporting changes in accelerometer-measured PA and SB during the COVID-19 pandemic is scarce [10, 11]. Thus, the present population-based study aimed to describe secular trends in daily steps, vigorous (VPA), moderate (MPA), MVPA, light physical activity (LPA), standing, and SB in three nationally representative cross-sectional samples of Finnish children and adolescents collected before the pandemic in 2016 and 2018, and after the pandemic in 2022. Primarily, the trends in PA, standing and SB for school grades, and for boys and girls separately were analyzed. Additionally, respective trends for weekdays and weekends were also assessed.

## Methods

The study is based on the Finnish school-aged physical activity (FSPA) Study, which is a national cross-sectional study on children’s and adolescents’ PA and SB. The study includes a web-based questionnaire and accelerometer measurements of PA, standing and SB for seven consecutive days, which form the basis for the present study.

Each study year the data collection was conducted from February to May. In 2016, the study sample included 3rd, 5th, 7th, and 9th graders attending the normal education in public schools [12]. In 2018 and 2022, the sample also included the 1st graders. In the Finnish school system, the respective mean age of pupils is generally 7-, 9-, 11-, 13-, and 15-years.

**Fig. 1:**
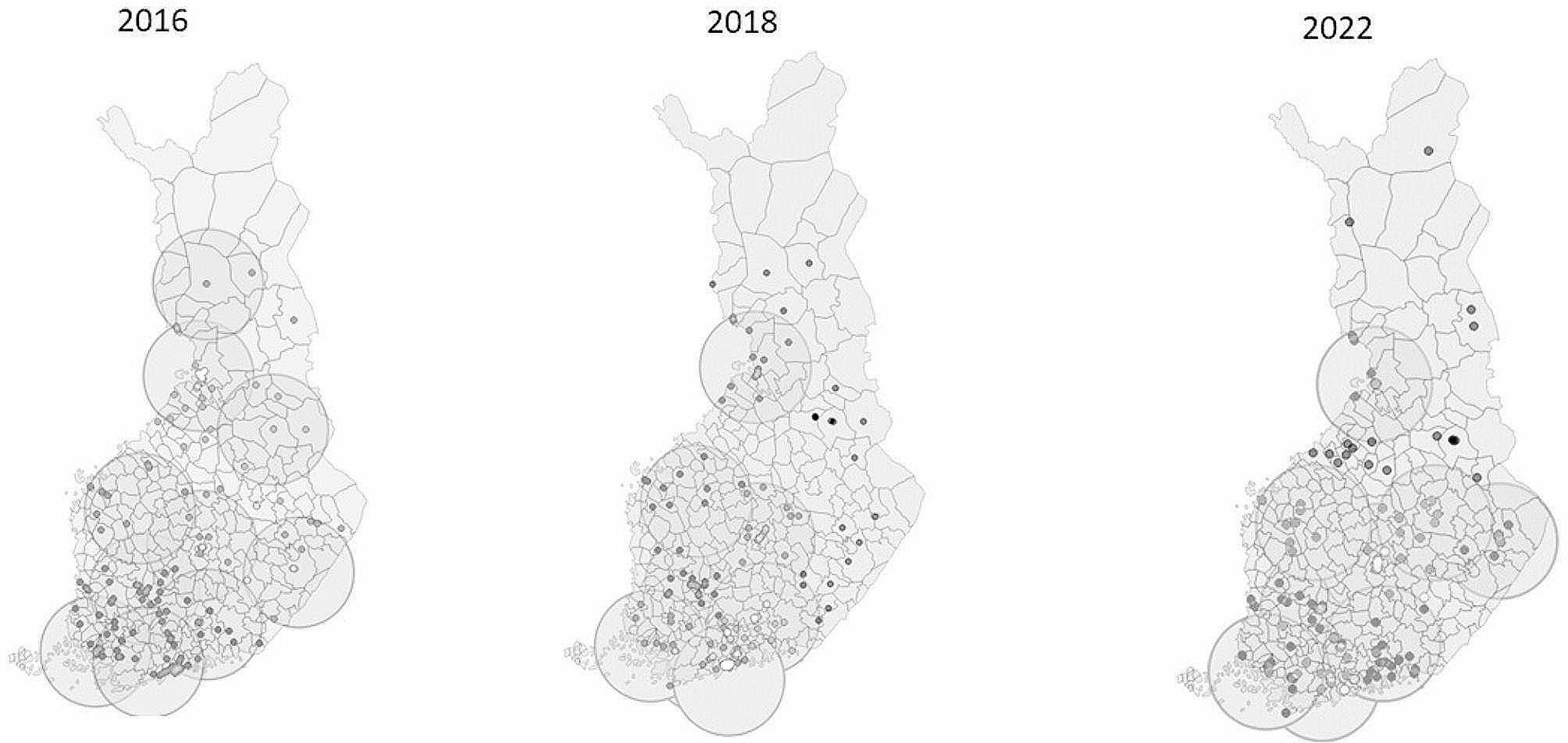
Locations of the regional organizations in 2016, 2018 and 2022. Small dots represent the participating schools and bigger circles the area of each regional organization executing the measurements

The FSPA Study was organized by the University of Jyväskylä, Finland, and the accelerometer measurements were conducted by the UKK Institute for Health Promotion Research, Tampere, Finland. For each study year, a stratified random sample of schools was drawn from the Statistics Finland database to form the study sample for a questionnaire-based survey. Schools were stratified according to ten different population areas: Capital city area and three different parts of Finland (South, Middle and North) categorized into big and small cities, and countryside. The accelerometer measurements were conducted in a sub-sample of the large survey. In each study year, there were approximately 10 regional organizations (Fig. 1) executing the measurements under the supervision of the UKK Institute. The inclusion criterion for schools to be included in the accelerometer study was a maximum of 100-km distance between the school and the regional organization [12]. The measurements for the schools that located further away from the organizations were conducted by the UKK Institute via mail.

First, a municipality level approval for conducting the study was applied for each school selected for the sample. Second, the principals of the schools selected to the accelerometer study decided whether the school was willing to participate the study and, which classes they invited to participate. Third, pupils of these classes received an information sheet of the study and an invitation to participate. Children younger than 15 years of age needed a written informed consent from their guardian to participate. Adolescents aged 15 year or older gave the consent on their own. If the responses of the municipalities and principals yielded a lack of participating schools from a certain population area, the sample was complemented by further sampling from the same population area. In 2016, a total of 176 of schools with 7.061 potential participants were willing to participate in the accelerometer study [12]. In 2018, the corresponding figures were 169 schools and 6.662 potential participants and, in 2022, 129 schools and 5.567 potential participants.

In 2016, the data was collected with UKK AM30 and RM42 accelerometers (UKK Terveyspalvelut Oy, Tampere, Finland), and in 2018 and 2022, only with UKK RM42. The RM42 accelerometer is an updated version of the AM30, which both employ the same sensor component (ADXL345; Analog Devices Inc, Norwood, MA, USA), collect the data with the same specifications, and analyze the data identically [12]. In 2016, the accelerometer was attached to an elastic belt and worn on the right side of the hip during waking hours for seven consecutive days. In 2018 and 2022, the accelerometer was worn 24/7. During waking hours, the accelerometer was worn on the right side of the hip similarly as in 2016. During the time-in-bed for sleeping, the accelerometer was moved to a wristband worn on the knuckle side of the non-dominant wrist. The location of the accelerometer was automatically recognized from the profile of detected changes in the device orientation in relation to the Earth’s gravity vector [13]. In the present study, time-in-bed was excluded from the analysis, and the participants who used the accelerometer for at least four days during a week with a minimum of 10 h daily waking wear time were included. The analysis for weekdays included at least three weekdays and that for weekends at least one weekend day with at least 10 h of daily waking wear time.

The analysis algorithms for PA, standing and SB have been validated for children, adolescents [12, 14] and adults [15, 16]. Raw triaxial acceleration data were collected at 100 Hz sampling frequency. The analysis was based on the mean amplitude deviation (MAD) of the acceleration signal analyzed in 6-s epochs and the angle for posture estimation (APE) [15, 16]. The MAD value corresponds to the intensity of PA, whereas the APE value indicates the body posture (i.e., lying, sitting, and standing). The MAD values were converted to metabolic equivalent (MET) values, and the epoch-wise MET values were further smoothed by calculating a one-minute exponential moving average. Using the smoothed MET values, the total PA was classified as LPA (1.5–2.9 MET), MPA (3.0-5.9 MET), or VPA (6 MET or more). MPA and VPA were also combined into MVPA. Lying, reclining, and sitting denoted SB [17], whereas standing was analyzed separately [16]. The step detection algorithm was based on the vertical component of the measured acceleration. The vertical component is band-pass filtered and positive values are integrated. When the integral value exceeds the specified limit, the step is detected. The step algorithm requires about 0.8 m/s walking speed to detect every step [16].

### Statistical analysis

The data from each study year were combined and analyzed with the same algorithms. Marginal means and mean differences in PA, standing and SB with 95% Confidence Intervals (CI) were determined using Multivariate regression analysis (GLM) adjusted for the accelerometer wear time during waking hours, school grade, and sex. All analyses were conducted by SPSS version 29 (IBM Corp. 2020, Armonk, NY).

## Results

In 2016, altogether 3.284 children and adolescents used the accelerometer to at least some extent and 3.057 of them met the criteria for sufficient use (at least four days, at least 10 h per day). In 2018 the corresponding numbers were 2.628 out of 2.782 participants and in 2022 1.395 out of 1.525 participants. Thus, the total sample of the present study comprised 7.080 participants. Sample size broken down by sex (57% were girls), school grade, and the study year are shown in Table 1. The participation was the highest in 2016 and the lowest in 2022. The analysis for weekdays included 7.143 participants in total (2016: 3.045; 2018: 2.686 and 2022: 1.412) and that for weekends 6.712 participants (2016: 2.786; 2018: 2.554 and 2022: 1.372).

**Table 1:** The number of participants in sex- and school-grade-groups

		1st grade	3rd grade	5th grade	7th grade	9th grade	Total
Boys	2016	-	407	420	265	183	1276*
	2018	251	384	275	147	126	1183
	2022	103	234	184	53	32	606
	Total	354	1025	879	465	341	3065
Girls	2016	-	511	497	436	321	1769*
	2018	249	439	353	204	200	1445
	2022	117	262	214	95	101	789
	Total	366	1212	1064	735	622	4003
Total	2016	-	919	921	703	509	3057*
	2018	500	823	628	351	326	2628
	2022	220	496	398	148	133	1395
	Total	720	2238	1947	1202	968	7080

*one boy and four girls had missing data for school grade

The participants wore the accelerometer on average 13–14 h per day during waking hours, and there were no systematic differences in wear times between the study years. (Supplementary Table 1). In each study year, the younger participants had a shorter mean wear time than the older ones.

**Fig. 2:**
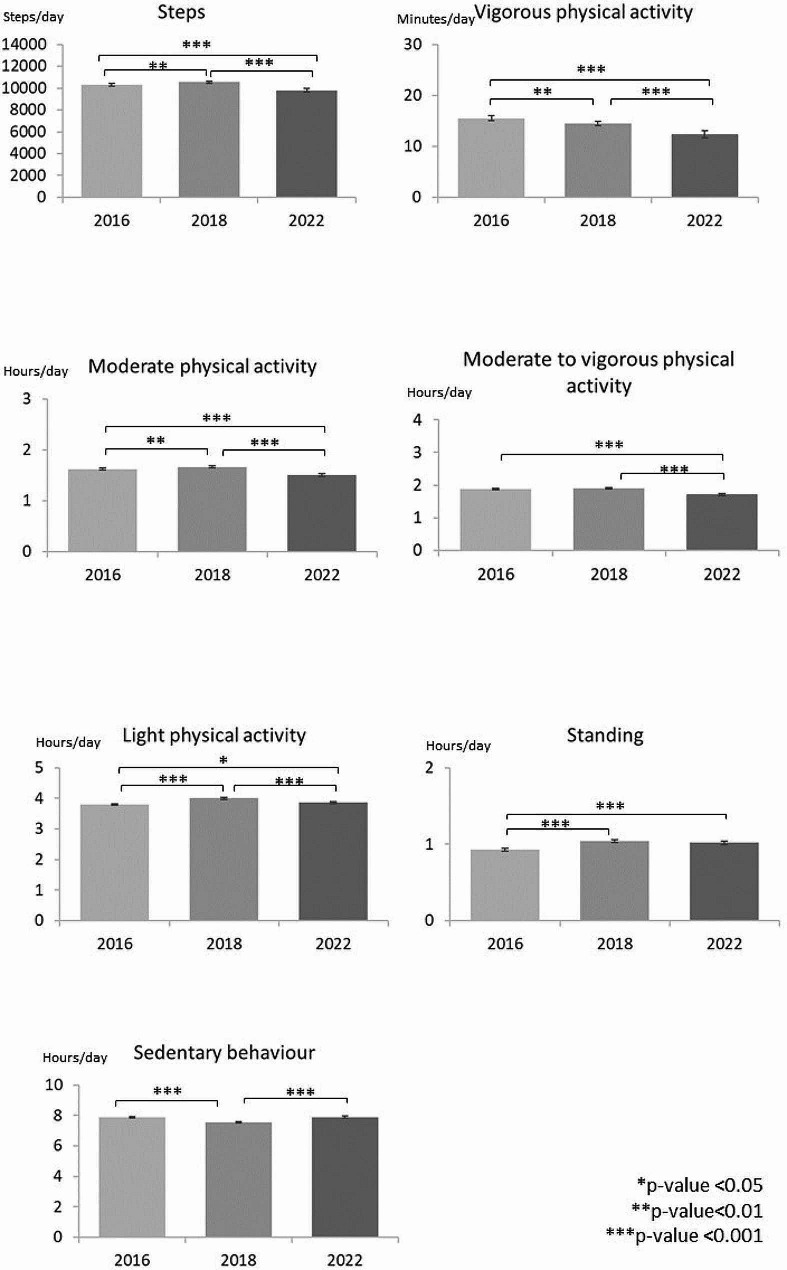
Differences in PA, standing and SB in 2016, 2018, and 2022

**Fig. 3:**
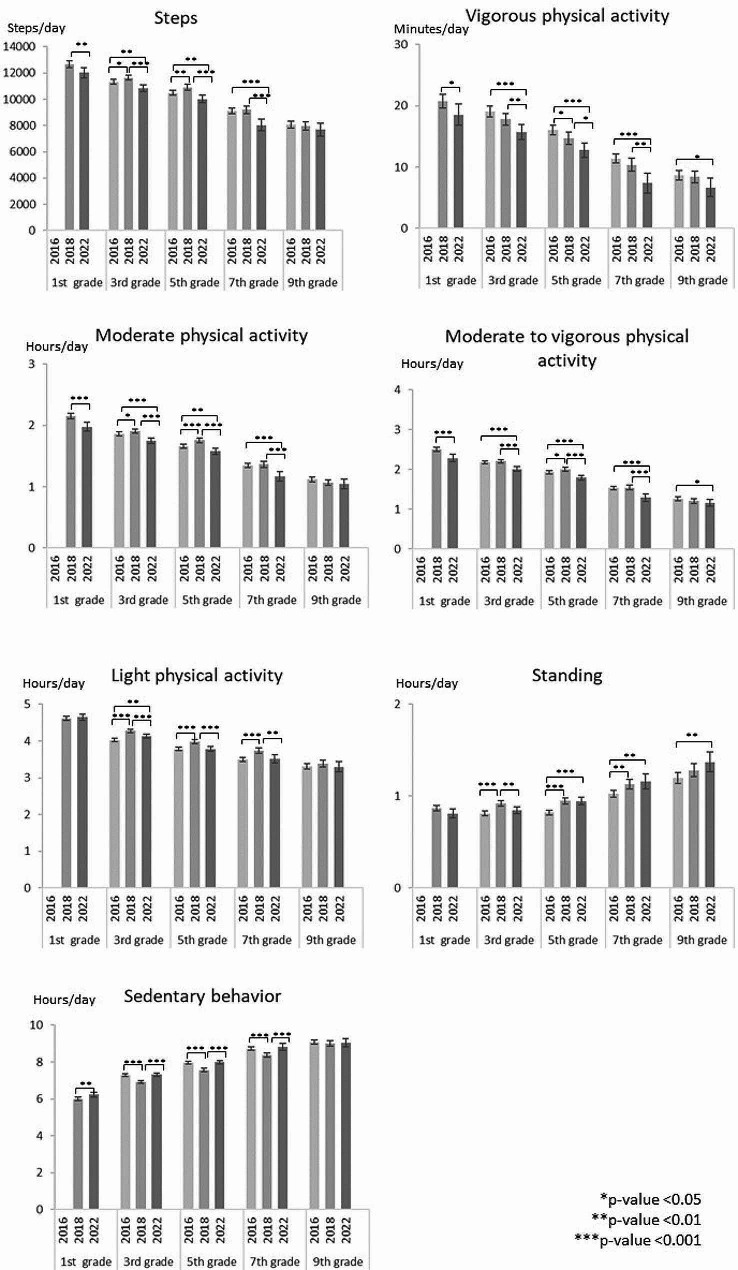
The school-grade-specific analysis of the differences in PA, standing and SB in 2016, 2018, and 2022

**Fig. 4:**
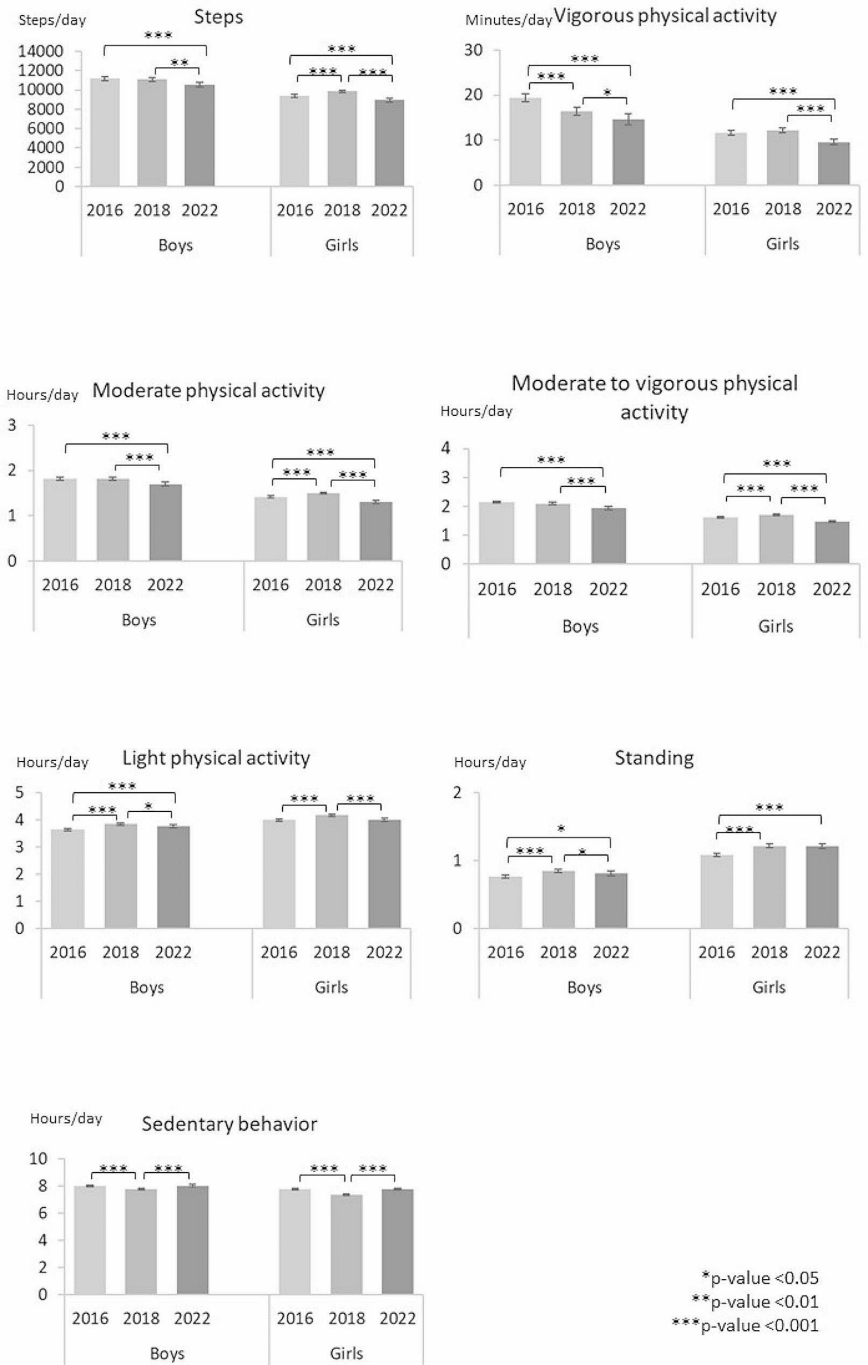
The sex-specific analysis of the differences in PA, standing and SB in 2016, 2018, and 2022

When the accelerometer wear time during waking hours, school grade and sex were adjusted for, the participants took on average 10.305 steps per day in 2016. In 2018 the corresponding number was 10.533 steps and in 2022 9.783, indicating a 7% decrease in the daily steps between 2018 and 2022 (Fig. 2, Supplementary Table 2). When the daily steps were analyzed according to school grade, there was a slight increase among the 3rd and 5^th,^ graders between 2016 and 2018, but a decrease thereafter. Among the 7th graders, there was no change in the daily steps between 2016 and 2018, but a decrease to 2022 (Fig. 3). Also, the daily steps of the 1st graders decreased from 2018 to 2022. The boys’ daily steps did not change between 2016 and 2018 but decreased to 2022. Among the girls, the daily steps increased from 2016 to 2018, but decreased to 2022 (Fig. 4). In each study year, children and adolescents took more steps on average during school days than during the weekend days (Supplementary figure). The daily steps remained unchanged on weekdays from 2016 to 2018 but decreased thereafter. On weekends, the daily steps increased between 2016 and 2018 and decreased thereafter.

The participants had on average 15.5 min VPA per day in 2016. In 2018 and 2022, the corresponding times were 14.5 min and 12.4 min, indicating a 7% decrease in daily VPA from 2016 to 2018 and further a 14% decrease to 2022 (Fig. 2, Supplementary Table 2). Among the 1st graders, the VPA time decreased from 2018 to 2022 (Fig. 3). Among the 3rd and 7th graders, the daily VPA time showed no change from 2016 to 2018 but decreased to 2022. Among the 5th graders, there was a decrease between 2016 and 2018 which continued to 2022. Among the 9th graders, there was no change in daily VPA time. Each study year, the boys accumulated on average more VPA per day than the girls (Fig. 4). However, among the boys the VPA time decreased between 2016 and 2018 continuing to 2022. Among the girls, there was no change from 2016 to 2018, but a decrease to 2022. Similar to the daily steps, the mean VPA time was higher on the weekdays than on weekends (Supplementary figure). Weekday activity declined between 2016 and 2018 continuing to 2022. The VPA time on weekend days declined only from 2018 to 2022.

Children and adolescents spent on average 1:37 (h: min) in MPA in 2016. In 2018, the corresponding time was 1:39 indicating a 2% increase (Fig. 1, Supplementary Table 2). In 2022, children and adolescents spent on average 9% less time in MPA than in 2018. In the school-grade specific analysis, the daily MPA time increased among the 3rd and 5th graders between 2016 and 2018, while there were no changes among the 7th and 9th graders (Fig. 3). The MPA time decreased among all other grades between 2018 and 2022, except the 9th graders. In each study year, boys accumulated on average more daily MPA than girls (Fig. 4). Among the boys, there was no change in MPA time from 2016 to 2018, but a decrease to 2022. Among the girls, the MPA time increased between 2016 and 2018, but decreased thereafter. Children and adolescents accumulated on average more MPA time on weekdays than on weekends (Supplementary figure). The MPA time on both weekdays and weekends increased between 2016 and 2018 but decreased to 2022.

When MPA and VPA were combined, children and adolescents spent on average 1:52 (h: min) per day in MVPA in 2016, 1:54 in 2018 and 1:42 in 2022 (Fig. 1, Supplementary Table 2). The daily MVPA time remained unchanged from 2016 to 2018 but declined by 11% thereafter. The corresponding trend was seen among the 1st, 3rd, and 7th graders, but among the 5th graders the daily MVPA time increased from 2016 to 2018 and declined thereafter (Fig. 3). Among the 9th graders the daily MVPA time declined only from 2018 to 2020. Boys showed a decreasing trend in the daily MVPA, but the change was statistically significant only between 2018 and 2020 (Fig. 4). Among the girls the daily MVPA time increased from 2016 to 2018 but decreased thereafter even to a lower level than in 2016.

Similar to daily steps, VPA, and MPA, also MVPA times were on average higher on weekdays than on weekends in each study year (Supplementary figure). Weekday MVPA times remained unchanged between 2016 and 2018, while on weekends the mean daily MVPA time increased. From 2018 to 2022, the daily MVPA time decreased both on weekdays and weekends.

Daily LPA time increased from 3:48 (h: min) in 2016 to 3:59 in 2018 indicating a 5% increase (Fig. 1, Supplementary Table 2). In 2022, the LPA time decreased on average by 3% being still on higher level than in 2016 (Fig. 3). Among the 3rd, 5th and 7th graders, the mean daily LPA time increased between 2016 and 2018 but decreased thereafter. Among the 9th graders, the daily LPA time remained unchanged. Both in boys and girls, the daily LPA times increased between 2016 and 2018 but decreased from 2018 to 2022 (Fig. 4). In each study year, the children and adolescents had on average more LPA time on weekdays than on weekends. The observed trends in LPA showed an increase from 2016 to 2018 and a decrease to 2022 both on weekdays and weekends (Supplementary figure).

In 2016, children and adolescents stood daily on average 55 min per day. In 2018, the daily standing time was 62 min, and in 2022 61 min indicating a 13% increase between 2016 and 2018 but no change thereafter (Fig. 1, Supplementary Table 2). In the school-grade-specific analysis, the 3rd graders showed an increase in daily standing time between 2016 and 2018 (Fig. 3), but a decrease to 2022. Among the 5th and 7th, graders, the standing time increased from 2016 to 2018, but did not change thereafter. In the sex-specific analysis, both boys and girls showed an increase in the daily standing time between 2016 and 2018. Thereafter the boys, but not girls, showed a decrease (Fig. 4). Correspondingly, standing time increased from 2016 to 2018 both on weekdays and weekends (Supplementary figure). In each study year, children and adolescents stood on average more on weekdays than on weekends.

The participants were sedentary on average 7:53 in 2016. In 2018 and 2022, the corresponding sedentary times were 7:33 and 7:54 (Fig. 1, Supplementary Table 2). These results indicated a 4% decrease between 2016 and 2018 and an 5% increase to 2022. When the daily SB time was analyzed according to school grade, among the 3rd, 5(th), and 7th graders there was a decrease between 2016 and 2018, but an increase to 2022 (Fig. 3). Also, the daily SB time of the 1st graders increased from 2018 to 2022. Among the 9th graders, there was no statistically significant change in the daily time. Both boys and girls showed a decrease in daily SB time from 2016 to 2018, and an equal increase from 2018 to 2022 (Fig. 4). In 2016, children and adolescents were on average more sedentary on weekends than on weekdays. In both 2018 and 2022, the mean daily SB time was equal on weekdays and weekends (Supplementary figure). Daily SB decreased between 2016 and 2018 but increased thereafter on both weekdays and weekends.

## Discussion

There was a declining trend in accelerometer-measured PA among Finnish children and adolescents from 2016 to 2022. The second data collection of the study in 2018 was conducted before the COVID-19 pandemic and the latest one in 2022 after the pandemic. Thus, the comparison of these two time points permits a reasonable estimation of the influence of the pandemic on PA, standing and SB. Both the decline in PA, regardless of intensity, and the increase in SB were evident between 2018 and 2022, which suggests at least some influence of the pandemic. During the pandemic time in Finland, in 2020 and 2021, the schools turned to distance learning, organized sports activities were closed for several months, and people were instructed to avoid social contacts. At the data collection in spring 2022, when the pandemic-related restrictions were no more active, PA and SB had not recovered to the pre-pandemic level. However, we cannot rule out the possibility that also non-pandemic causes could have influenced to the results obtained.

The findings of the present study are in line with Wunsch et al. [11] who reported that PA in children and adolescents decreased during the COVID-19 pandemic, and the decrease was independent of sex when assessed both in terms of self-reported and device-based measurements. Also, a recent review by Rossi et al. [10] reported a decrease in total PA during the pandemic, while an increase was observed in unstructured and outdoor activities.

The number of daily steps declined on average by 751 steps from 2018 to 2022, meaning that nearly 300.000 steps were lost every year. In a recent systematic review [8], the daily steps in children younger than 11 years declined by 823 steps/decade between 1995 and 2017. In the present study, the mean decline among the 3rd graders (on average 9-year-old) was 469 steps/day in six years corresponding to 782 steps/decade. The greatest decline was observed among the 7th graders, whose daily steps declined by 1.187 steps between 2018 and 2022. In the review by Conger et al. [8], the daily steps in adolescents declined by 1.497 steps/decade. If these declines are interpreted on a yearly basis, the present study showed a larger decline. One reason for the difference may be that the review [8] was conducted before the COVID-19 pandemic when the pandemic-related restrictions had not yet affected the activity levels. The other reasons for the differences may be due to different measurement methods to assess steps and differences in study populations.

A national study conducted in Norway reported tendencies towards decreasing PA levels among children and adolescents between 2005, 2011, and 2018 [18]. The most pronounced change was observed among the 9-year-old boys, whose daily MVPA time declined by 10 min during 13 years [18]. In the present study, the youngest participants (the 3rd and 5th graders) showed a slight increase in their PA levels between 2016 and 2018, but a clear decline thereafter. In line with the step-related results, the most pronounced decline in MVPA was found among the 7th graders, whose mean daily MVPA declined by 14 min between 2018 and 2022. According to Garcia-Hermoso et al. [19], VPA is particularly important for the cardiometabolic health of children and adolescents and its role should be strengthened further in the PA recommendations. Declined MPA and VPA may result in deteriorated physical fitness as demonstrated earlier in young Finnish men [20].

A meta-regression analysis on school-day PA showed that MVPA declined between 2003 and 2010, plateaued 2010–2015, and increased 2015–2019 [9]. The present study showed a steady MVPA level from 2016 to 2018, but a subsequent decrease. We did not separate school hours from the total PA, but our previous detailed analysis revealed that among the less active children and adolescents most of their daily PA accumulated during the school hours [12]. Thus, schools provide an important setting for promoting PA [21]. Most children and adolescents spend a lot of their waking hours at school, schools have the relevant infrastructure, they possess a variety of opportunities to be active during recess and lessons and provide also out-school opportunities for PA through active transportation and after-school activities.

Changes in PA levels are associated with changes in cardiometabolic risk factors [22]. Decreased PA is associated with unfavorable changes in body mass index, insulin, glucose, and high-density cholesterol [22]. Although decreasing PA observed in the present study is based on cross-sectional population-level findings, it may reflect unfavorable changes in corresponding health indicators as reported by Aira et al. [22]. Maintenance of high PA levels, especially MVPA, in young adulthood, is a critical point for maintaining metabolic health later in adulthood [23]. Thus, adolescence and young adulthood are opportune periods for interventions to ensure adequate accumulation of daily MVPA.

Sex-specific analysis of the present study revealed that girls accumulated on average more LPA than boys in each study year, while the amounts of VPA, MPA, and MVPA were consistently higher among the boys. Previously girls have been reported to have less MVPA than boys [5, 24], while there have been no sex-differences in LPA [24]. In the present study, LPA showed a slight increase among both boys and girls from 2016 to 2018, but a decline thereafter. Also, MPA, VPA, MVPA and daily steps declined among both sexes from 2018 to 2022, the decline in steps being slightly greater in girls than in boys (931 vs. 567 steps). Previous studies have reported inconsistent findings regarding sex-specific trends in PA. Conger et al. [8] reported a similar decline in PA among adolescent boys and girls, while Guthold et al. [5] found an increase in PA among boys and no changes among the girls during 2001–2016. Steene-Johannessen et al. [18] reported a greater decline in MVPA among boys than girls during 2005–2018 (9.7 min/d vs.3.2 min/d). In the present study daily MVPA declined 12 min among the boys and 9 min among the girls from 2016 to 2022.

The participants of the present study were on average more active on weekdays than on weekends, irrespective of PA intensity. This was evident in each study year concurring with previous findings [18, 25]. Kallio et al. [25] reported on average 14 min/d difference in MVPA between weekdays and weekend days, favoring weekdays. The temporal trend in LPA of the present study was similar for weekdays and weekends. Weekday VPA declined from 2016 to 2018 and, continuing to 2022, but on weekends VPA declined only from 2018 to 2022. MPA in turn, showed an increase between 2016 and 2018 and declined thereafter both on weekdays and weekends. Steene-Johannessen et al. [18] reported somewhat larger relative declines in PA on weekends than on weekdays, although the declines were age-specific.

Previous studies of time trends of overall PA among children and adolescents have focused mostly on MVPA. To our knowledge, no previous studies have reported trends of both accelerometer-measured SB and standing over several years among school-aged children and adolescents. In the present study, SB showed a slight decrease between 2016 and 2018 and a subsequent increase in all other age groups but the 9th graders. Daily standing time showed a clear increase between 2016 and 2018 and a stable trend thereafter. Previous studies have reported increases both in accelerometer-measured SB [25–27] and self-reported screen time [28], computer use and total sitting time [29, 30] among children and adolescents, but the trends in standing have not been reported. Assessing trends in standing would also be important since sitting less and standing more may be an effective way to improve health indicators among adolescents, regardless of their MVPA [31].

An increase in MPA, LPA, and daily steps and a decrease in SB seen in the present study from 2016 to 2018 may be partly due to a national action program (Schools on the move) established in 2010. The program aims to establish a physically active culture in comprehensive schools, and currently the program covers more than 90% of Finnish schools [32]. However, the latest measurements of the present study showed a clear decrease in daily PA regardless of intensity and an increase in SB, which may reflect, at least partly, the effects of COVID-19 pandemic occurring between the data collections in 2018 and 2022.

There are a few limitations that need to be acknowledged when interpreting the present findings. The participants wore the accelerometer on their hip, and thus PA solely due to upper body movements, without hip movement, load-carrying in place, and other non-bipedal activities may be missed, or their intensity underestimated. The accelerometer was not used during water-based activities like showering, bathing, and swimming. Also, the contexts or types of PA, standing and SB could not be detected. Accelerometer wearing protocol and model changed during the measurements which can be regarded as a limitation. However, both accelerometers (UKK AM30 and UKK RM42) use the same sensor component. The data was processed and analyzed similarly, and the accelerometer model did not affect the results. The different wearing time influenced the results and therefore the wear time during waking hours was controlled for in the analysis. Thus, no differential misclassification over time can be expected. Further, the number of participating schools together with that of children and adolescents declined over time, especially between 2018 and 2022, which may have affected the results. The decline of participation rate was seen at all levels of recruitment (municipal, school, and participant levels), which may be due to several challenges the society was facing in 2022 (for example recovery from COVID-19 pandemic and teachers’ strike in Finland at late spring 2022). Lack of relevant descriptive data (i.e., anthropometric data and socioeconomic status) is also a limitation of the study.

The strengths of the study include a large nationally representative population-based sample, a wide age range of participants of both sexes, and the analysis of PA, standing and SB with validated accelerometer algorithms [14–16]. The ability to separate standing from SB and PA is a unique feature of this analysis method. To plan actions to promote PA and to evaluate their effectiveness and the development of PA, standing and SB further, it is important to monitor population levels of these behaviors continuously and systematically with the same device-based methods [8, 18]. FSPA Study is a regularly conducted nationwide study that offers a possibility to monitor accelerometer-measured trends in PA, standing and SB regularly also in the future.

## Conclusions

This study provided novel, accelerometer-measured data on the development of PA, standing and SB among school-aged children and adolescents from 2016 to 2022. Besides commonly reported PA parameters, the study provided data on standing and SB which have not been measured in many previous studies. The main finding of the study indicates that PA decreased, and sedentariness increased during the COVID-19 pandemic and no recovery was seen until spring 2022. Actions are needed to turn the decreasing trend of PA among children and adolescents to a more active direction. PA levels were especially low among adolescents and girls. Thus, promotion of PA and reduction of SB should be targeted particularly at these groups. Weekends were physically less active than the weekdays, which offers great potential to increase activity levels on weekend days. Future studies are needed to find feasible and appropriate ways to increase PA and decrease SB among adolescents, especially in girls and on weekends.

### Electronic supplementary material

Below is the link to the electronic supplementary material.

Supplementary Material 1

Supplementary Material 2. Supplementary figure PA and SB on weekdays and weekend days in 2016, 2018, and 2022.

Supplementary Material 3

## Data Availability

The datasets analyzed in the present study are not publicly available due to ethical restrictions (the Ethics Committee of the University of Jyväskylä), but more detailed information on the data is available from TV on reasonable request.

## Abbreviations

APE: Angle for posture estimation
CI: Confidence Interval
FSPA: Finnish school-aged physical activity
LPA: Light physical activity
MAD: Mean amplitude deviation
MET: Metabolic equivalent
MPA: Moderate physical activity
MVPA: Moderate-to-vigorous physical activity
PA: Physical activity
SB: Sedentary behavior
VPA: Vigorous physical activity

## References

[1] Piercy KL, Troiano RP, Ballard RM, Carlson SA, Fulton JE, Galuska DA, George SM, Olson RD (2018). The physical activity guidelines for americans. JAMA.

[2] Carson V, Hunter S, Kuzik N, Gray CE, Poitras VJ, Chaput JP, Saunders TJ, Katzmarzyk PT, Okely AD, Connor Gorber S (2016). Systematic review of sedentary behaviour and health indicators in school-aged children and youth: an update. Appl Physiol Nutr Metab.

[3] Wu XY, Han LH, Zhang JH, Luo S, Hu JW, Sun K (2017). The influence of physical activity, sedentary behavior on health-related quality of life among the general population of children and adolescents: a systematic review. PLoS ONE.

[4] Poitras VJ, Gray CE, Borghese MM, Carson V, Chaput JP, Janssen I, Katzmarzyk PT, Pate RR, Connor Gorber S, Kho ME (2016). Systematic review of the relationships between objectively measured physical activity and health indicators in school-aged children and youth. Appl Physiol Nutr Metab.

[5] Guthold R, Stevens GA, Riley LM, Bull FC (2020). Global trends in insufficient physical activity among adolescents: a pooled analysis of 298 population-based surveys with 1.6 million participants. Lancet Child Adolesc Health.

[6] Steene-Johannessen J, Hansen BH, Dalene KE, Kolle E, Northstone K, Moller NC, Grontved A, Wedderkopp N, Kriemler S, Page AS (2020). Variations in accelerometry measured physical activity and sedentary time across Europe - harmonized analyses of 47,497 children and adolescents. Int J Behav Nutr Phys Act.

[7] Corder K, Winpenny E, Love R, Brown HE, White M, Sluijs EV (2019). Change in physical activity from adolescence to early adulthood: a systematic review and meta-analysis of longitudinal cohort studies. Br J Sports Med.

[8] Conger SA, Toth LP, Cretsinger C, Raustorp A, Mitas J, Inoue S, Bassett DR (2022). Time trends in Physical Activity using Wearable devices: a systematic review and Meta-analysis of studies from 1995 to 2017. Med Sci Sports Exerc.

[9] Weaver RG, Tassitano RM, Tenorio MCM, Brazendale K, Beets MW (2021). Temporal trends in Children’s School Day Moderate to Vigorous Physical activity: a systematic review and Meta-regression analysis. J Phys Act Health.

[10] Rossi L, Behme N, Breuer C. Physical activity of children and adolescents during the COVID-19 Pandemic-A scoping review. Int J Environ Res Public Health 2021, 18(21). 10.3390/ijerph182111440 PMC8583307 34769956

[11] Wunsch K, Kienberger K, Niessner C. Changes in physical activity patterns due to the Covid-19 pandemic: a systematic review and Meta-analysis. Int J Environ Res Public Health 2022, 19(4). 10.3390/ijerph19042250 PMC8871718 35206434

[12] Jussila AM, Husu P, Vaha-Ypya H, Tokola K, Kokko S, Sievanen H, Vasankari T. Accelerometer-measured physical activity levels and patterns vary in an age- and sex-dependent fashion among Finnish children and adolescents. Int J Environ Res Public Health 2022, 19(11). 10.3390/ijerph19116950 PMC9180141 35682533

[13] Husu P, Tokola K, Vähä-Ypyä H, Sievänen H, Suni J, Heinonen O, Heiskanen J, Kaikkonen K, Savonen K, Kokko S, Vasankari T (2021). Physical activity, sedentary behavior and time in bed among Finnish adults measured 24/7 by triaxial accelerometry. J Meas Phys Behav.

[14] Aittasalo M, Jussila AM, Tokola K, Sievanen H, Vaha-Ypya H, Vasankari T (2019). Kids out; evaluation of a brief multimodal cluster randomized intervention integrated in health education lessons to increase physical activity and reduce sedentary behavior among eighth graders. BMC Public Health.

[15] Vaha-Ypya H, Vasankari T, Husu P, Manttari A, Vuorimaa T, Suni J, Sievanen H (2015). Validation of cut-points for evaluating the intensity of physical activity with Accelerometry-Based Mean Amplitude deviation (MAD). PLoS ONE.

[16] Vaha-Ypya H, Husu P, Suni J, Vasankari T, Sievanen H (2018). Reliable recognition of lying, sitting, and standing with a hip-worn accelerometer. Scand J Med Sci Sports.

[17] Tremblay MS, Aubert S, Barnes JD, Saunders TJ, Carson V, Latimer-Cheung AE, Chastin SFM, Altenburg TM, Chinapaw MJM, Participants STCP (2017). Sedentary Behavior Research Network (SBRN) - terminology Consensus Project process and outcome. Int J Behav Nutr Phys Act.

[18] Steene-Johannessen J, Anderssen SA, Kolle E, Hansen BH, Bratteteig M, Dalhaug EM, Andersen LB, Nystad W, Ekelund U, Dalene KE (2021). Temporal trends in physical activity levels across more than a decade - a national physical activity surveillance system among Norwegian children and adolescents. Int J Behav Nutr Phys Act.

[19] Garcia-Hermoso A, Ezzatvar Y, Ramirez-Velez R, Olloquequi J, Izquierdo M (2021). Is device-measured vigorous physical activity associated with health-related outcomes in children and adolescents? A systematic review and meta-analysis. J Sport Health Sci.

[20] Santtila M, Pihlainen K, Koski H, Vasankari T, Kyrolainen H (2018). Physical fitness in Young men between 1975 and 2015 with a focus on the years 2005–2015. Med Sci Sports Exerc.

[21] Fenesi B, Graham JD, Crichton M, Ogrodnik M, Skinner J. Physical activity in High School classrooms: a Promising Avenue for Future Research. Int J Environ Res Public Health 2022, 19(2). 10.3390/ijerph19020688 PMC8776126 35055510

[22] Aira T, Kokko SP, Heinonen OJ, Korpelainen R, Kotkajuuri J, Parkkari J, Savonen K, Toivo K, Uusitalo A, Valtonen M (2023). Longitudinal physical activity patterns and the development of cardiometabolic risk factors during adolescence. Scand J Med Sci Sports.

[23] Nagata JM, Cortez CA, Dooley EE, Iyer P, Ganson KT, Pettee Gabriel K (2022). Moderate-to-vigorous intensity physical activity among adolescents in the USA during the COVID-19 pandemic. Prev Med Rep.

[24] Kretschmer L, Salali GD, Andersen LB, Hallal PC, Northstone K, Sardinha LB, Dyble M, Bann D (2023). International Children’s Accelerometry database C: gender differences in the distribution of children’s physical activity: evidence from nine countries. Int J Behav Nutr Phys Act.

[25] Kallio J, Hakonen H, Syvaoja H, Kulmala J, Kankaanpaa A, Ekelund U, Tammelin T (2020). Changes in physical activity and sedentary time during adolescence: gender differences during weekdays and weekend days. Scand J Med Sci Sports.

[26] van Ekris E, Wijndaele K, Altenburg TM, Atkin AJ, Twisk J, Andersen LB, Janz KF, Froberg K, Northstone K, Page AS (2020). Tracking of total sedentary time and sedentary patterns in youth: a pooled analysis using the International Children’s Accelerometry database (ICAD). Int J Behav Nutr Phys Act.

[27] Velazquez-Romero MJ, Padilla-Moledo C, Segura-Jimenez V, Sanchez-Oliva D, Fernandez-Santos JR, Senin-Calderon C, Grao-Cruces A (2021). Trends of Sedentary Time and Domain-Specific Sedentary Behavior in Spanish Schoolchildren. Res Q Exerc Sport.

[28] Cui Z, Hardy LL, Dibley MJ, Bauman A (2011). Temporal trends and recent correlates in sedentary behaviours in Chinese children. Int J Behav Nutr Phys Act.

[29] Yang L, Cao C, Kantor ED, Nguyen LH, Zheng X, Park Y, Giovannucci EL, Matthews CE, Colditz GA, Cao Y (2019). Trends in Sedentary Behavior among the US Population, 2001–2016. JAMA.

[30] Ryu S, Kim H, Kang M, Pedisic Z, Loprinzi PD (2019). Secular trends in Sedentary Behavior among High School students in the United States, 2003 to 2015. Am J Health Promot.

[31] Moura BP, Rufino RL, Faria RC, Sasaki JE, Amorim PRS. Can Replacing Sitting Time with Standing Time Improve Adolescents’ Cardiometabolic Health? Int J Environ Res Public Health 2019, 16(17). 10.3390/ijerph16173115 PMC6747710 31461890

[32] Schools on the move -program. https://schoolsonthemove.fi/ (Accessed on March 28th, 2024).

