# Supplementary material for: Physical activity has decreased in Finnish children and adolescents from 2016 to 2022
Source: BMC Public Health. 2024 May 18;24:1343. doi: 10.1186/s12889-024-18854-7 (PMC11102264; doi:10.1186/s12889-024-18854-7)
Supplement: Supplementary file 1 — Supplementary Material 1 [file 12889_2024_18854_MOESM1_ESM.docx]

Supplementary table 1. The accelerometer wear times during waking hours in 2016, 2018 and 2022 according to sex and school grade.

|  |  | 1^st^ grade | 3^rd^ grade | 5^th^ grade | 7^th^ grade | 9^th^ grade |
| --- | --- | --- | --- | --- | --- | --- |
| Boys | 2016 |  | 14.4 | 14.6 | 14.6 | 14.8 |
|  | 2018 | 14.1 | 14.5 | 14.6 | 15.0 | 15.0 |
|  | 2022 | 14.1 | 14.3 | 14.6 | 14.9 | 15.1 |
|  | Total | 14.1 | 14.4 | 14.6 | 14.4 | 15.0 |
|  |  |  |  |  |  |  |
| Girls | 2016 |  | 14.3 | 14.4 | 14.7 | 14.8 |
|  | 2018 | 13.9 | 14.2 | 14.5 | 14.8 | 14.9 |
|  | 2022 | 13.8 | 14.1 | 14.4 | 14.9 | 15.0 |
|  | Total | 13.8 | 14.2 | 14.4 | 14.8 | 14.9 |
