# Supplementary figures and images for: Physical activity has decreased in Finnish children and adolescents from 2016 to 2022
Source: BMC Public Health. 2024 May 18;24:1343. doi: 10.1186/s12889-024-18854-7 (PMC11102264; doi:10.1186/s12889-024-18854-7)

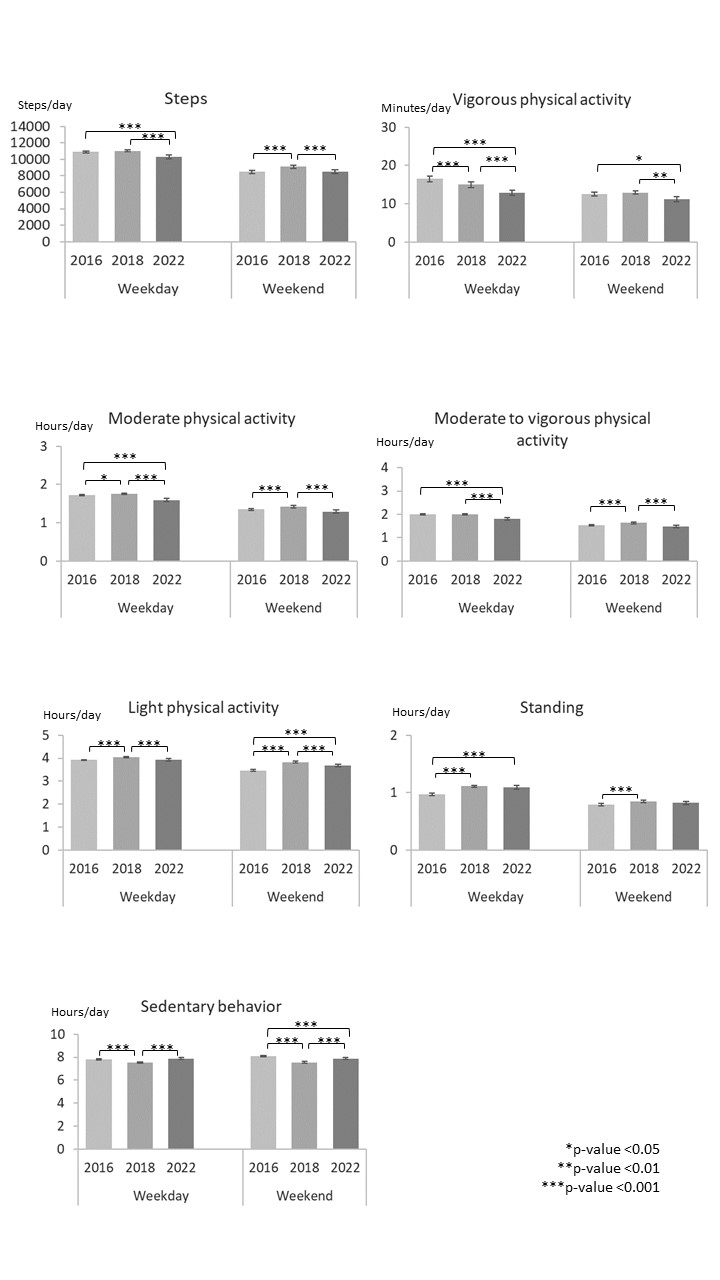

Supplement: Supplementary file 2 — Supplementary Material 2. Supplementary figure PA and SB on weekdays and weekend days in 2016, 2018, and 2022. [file 12889_2024_18854_MOESM2_ESM.jpg]
