# Supplementary material for: Physical activity has decreased in Finnish children and adolescents from 2016 to 2022
Source: BMC Public Health. 2024 May 18;24:1343. doi: 10.1186/s12889-024-18854-7 (PMC11102264; doi:10.1186/s12889-024-18854-7)
Supplement: Supplementary file 3 — Supplementary Material 3 [file 12889_2024_18854_MOESM3_ESM.docx]

Supplementary table 2. Daily number of steps, the amount of different PA intensities, standing and sedentary behavior in 2016, 2018 and 2022 according to school grade, sex and weekdays vs. weekends and relative changes between the study years.

|  | 2016 | | 2018 | | 2022 | | relative change 2016 vs. 2018 | relative change 2018 vs. 2022 | relative change 2016 vs. 2022 | significance of the change 2016 vs. 2018 | significance of the change 2018 vs. 2022 | significance of the change 2016 vs. 2022 |
| --- | --- | --- | --- | --- | --- | --- | --- | --- | --- | --- | --- | --- |
|  | mean | 95% CI | mean | 95% CI | mean | 95% CI |  |  |  | p-value* | p-value* | p-value* |
| Total |  |  |  |  |  |  |  |  |  |  |  |  |
| Steps (number/day) | 10305 | 10191-10419 | 10533 | 10420-10647 | 9783 | 9627-9939 | 2 % | -7 % | -5 % | 0.005 | <0.001 | <0.001 |
| Vigorous PA (h:min/day) | 0:15 | 0:15-0:16 | 0:14 | 0:13-0:14 | 0:12 | 0:11-0:13 | -7 % | -14 % | -20 % | 0.002 | <0.001 | <0.001 |
| Moderate PA (h:min/day) | 1:37 | 1:35-1:38 | 1:39 | 1:38-1:40 | 1:30 | 1:28-1:32 | 2 % | -9 % | -7 % | 0.001 | <0.001 | <0.001 |
| MVPA (h:min/day) | 1:52 | 1:51-1:54 | 1:54 | 1:52-1:55 | 1:42 | 1:40-1:44 | 2 % | -11 % | -9 % | 0.113 | <0.001 | <0.001 |
| Light PA (h:min/day) | 3:48 | 3:46-3:49 | 3:59 | 3:58-4:01 | 3:51 | 3.49-3:53 | 5 % | -3 % | 1 % | <0.001 | <0.001 | 0.012 |
| Standing (h:min/day) | 0:55 | 0:54-0:56 | 1:02 | 1:01-1:03 | 1:01 | 0:59-1:02 | 13 % | -2 % | 11 % | <0.001 | 0.180 | <0.001 |
| SB (h:min/day) | 7:52 | 7:50-7:55 | 7:32 | 7:30-3:35 | 7:53 | 7:50-7:57 | -4 % | 5 % | 0 % | <0.001 | <0.001 | 0.724 |
| 1st grade |  |  |  |  |  |  |  |  |  |  |  |  |
| Steps (number/day) |  |  | 12650 | 12398-12901 | 12019 | 11640-12398 |  | -5 % |  |  | 0.007 |  |
| Vigorous PA (h:min/day) |  |  | 0:20 | 0:19-0:21 | 0:18 | 0:16-0:20 |  | -10 % |  |  | 0.038 |  |
| Moderate PA (h:min/day) |  |  | 2:09 | 2:06-2:12 | 1:58 | 1:54-2:03 |  | -9 % |  |  | <0.001 |  |
| MVPA (h:min/day) |  |  | 2:30 | 0:26-2:33 | 2:17 | 2:11-2:22 |  | -9 % |  |  | <0.001 |  |
| Light PA (h:min/day) |  |  | 4:36 | 4:33-4:40 | 4:38 | 4:34-4:43 |  | 1 % |  |  | 0.463 |  |
| Standing (h:min/day) |  |  | 0:52 | 0:50-0:54 | 0:48 | 0:45-0:51 |  | -8 % |  |  | 0.054 |  |
| SB (h:min/day) |  |  | 6:00 | 5:54-6:05 | 6:14 | 6:06-6:21 |  | 4 % |  |  | 0.003 |  |
| 3rd grade |  |  |  |  |  |  |  |  |  |  |  |  |
| Steps (number/day) | 11322 | 11130-11515 | 11640 | 11437-11843 | 10853 | 10592-11115 | 3 % | -7 % | -4 % | 0.026 | <0.001 | 0.005 |
| Vigorous PA (h:min/day) | 0:19 | 0:18-0:19 | 0:17 | 0:16-0:18 | 0:15 | 0:14-0:16 | -11 % | -12 % | -21 % | 0.063 | 0.008 | <0.001 |
| Moderate PA (h:min/day) | 1:51 | 1:49-1:53 | 1:54 | 1:52-1:56 | 1:44 | 1:42-1:47 | 3 % | -9 % | -6 % | 0.032 | <0.001 | <0.001 |
| MVPA (h:min/day) | 2:10 | 2.08-2:13 | 2:12 | 2:09-2:15 | 2:00 | 1:57-2:03 | 2 % | -9 % | -8 % | 0.304 | <0.001 | <0.001 |
| Light PA (h:min/day) | 4:01 | 3:59-4:04 | 4:16 | 4:13-4:18 | 4:07 | 4:04-4:11 | 6 % | -4 % | 2 % | <0.001 | <0.001 | 0.003 |
| Standing (h:min/day) | 0:48 | 0:47-0:50 | 0:55 | 0:53-0:56 | 0:50 | 0:48-0:52 | 15 % | -9 % | 4 % | <0.001 | 0.001 | 0.147 |
| SB (h:min/day) | 7:17 | 7:13-7:21 | 6:54 | 6:49-6:58 | 7:19 | 7:13-7:24 | -5 % | 6 % | 0 % | <0.001 | <0.001 | 0.639 |
| 5th grade |  |  |  |  |  |  |  |  |  |  |  |  |
| Steps (number/day) | 10479 | 10291-10667 | 10909 | 10681-11137 | 10009 | 9723-10294 | 4 % | -8 % | -4 % | 0.004 | <0.001 | 0.007 |
| Vigorous PA (h:min/day) | 0:16 | 0:15-0:16 | 0:14 | 0:13-0:15 | 0:12 | 0:11-0:13 | -13 % | -14 % | -25 % | 0.037 | 0.013 | <0.001 |
| Moderate PA (h:min/day) | 1:39 | 1:37-1:41 | 1:45 | 1:43-4:48 | 1:34 | 1:31-1:37 | 6 % | -10 % | -5 % | <0.001 | <0.001 | 0.006 |
| MVPA (h:min/day) | 1:55 | 1:53-1:58 | 2:00 | 1:57-2:03 | 1:47 | 1:43 | 4 % | -11 % | -7 % | 0.018 | <0.001 | <0.001 |
| Light PA (h:min/day) | 3:47 | 3:44-3:49 | 3:59 | 3:56-4:02 | 3:46 | 3:42-3:50 | 5 % | -5 % | 0 % | <0.001 | <0.001 | 0.937 |
| Standing (h:min/day) | 0:49 | 0:47-0:50 | 0:57 | 0:55-0:59 | 0:56 | 0:54-0:59 | 16 % | -2 % | 14 % | <0.001 | 0.887 | <0.001 |
| SB (h:min/day) | 7:58 | 7:53-8:02 | 7:33 | 7:28-7:38 | 7:59 | 7:52-8:05 | -5 % | 6 % | 0 % | <0.001 | <0.001 | 0.812 |
| 7th grade |  |  |  |  |  |  |  |  |  |  |  |  |
| Steps (number/day) | 9106 | 8900-9311 | 9216 | 829-9502 | 8028 | 7587-8470 | 1 % | -13 % | -12 % | 0.537 | <0.001 | <0.001 |
| Vigorous PA (h:min/day) | 0:11 | 0:10-0:12 | 0:10 | 0:09-0:11 | 0:07 | 0:05-0:09 | -9 % | -30 % | -36 % | 0.113 | 0.002 | <0.001 |
| Moderate PA (h:min/day) | 1:20 | 1:18-1:23 | 1:22 | 1:19-1:25 | 1:10 | 1:05-1:14 | 3 % | -15 % | -13 % | 0.519 | <0.001 | <0.001 |
| MVPA (h:min/day) | 1:32 | 1:29-1:34 | 1:32 | 1:28-1:36 | 1:17 | 1:12-1:23 | 0 % | -16 % | -16 % | 0.948 | <0.001 | <0.001 |
| Light PA (h:min/day) | 3:29 | 3:26-3:33 | 3:44 | 3:40-3:48 | 3:30 | 3:23-3:37 | 7 % | -6 % | 0 % | <0.001 | 0.001 | 0.852 |
| Standing (h:min/day) | 1:01 | 0:59-1:04 | 1:07 | 1:04-1:11 | 1:09 | 1:04-1:14 | 10 % | 3 % | 13 % | 0.002 | 0.531 | 0.004 |
| SB (h:min/day) | 8:43 | 8:37-8:48 | 8:22 | 8:15-8:29 | 8:49 | 8:38-9:00 | -4 % | 5 % | 1 % | <0.001 | <0.001 | 0.330 |
| 9th grade |  |  |  |  |  |  |  |  |  |  |  |  |
| Steps (number/day) | 8067 | 7812-9323 | 7957 | 7643-8271 | 7699 | 7202-8195 | -1 % | -3 % | -5 % | 0.588 | 0.382 | 0.188 |
| Vigorous PA (h:min/day) | 0:08 | 0:07-0:09 | 0:08 | 0:07-0:09 | 0:06 | 0:05-0:08 | 0 % | -25 % | -25 % | 0.685 | 0.063 | 0.024 |
| Moderate PA (h:min/day) | 1:07 | 1:04-1:09 | 1:04 | 1:01-1:06 | 1:02 | 0:58-1:07 | -4 % | -3 % | -7 % | 0.095 | 0.651 | 0.091 |
| MVPA (h:min/day) | 1:15 | 1:13-1:18 | 1:12 | 1:09-1:15 | 1:09 | 1:04-1:14 | -4 % | -4 % | -8 % | 0.122 | 0.357 | 0.036 |
| Light PA (h:min/day) | 3:19 | 3:14-3:23 | 3:22 | 3:17-3:28 | 3:17 | 3:09-3:26 | 2 % | -2 % | -1 % | 0.269 | 0.332 | 0.825 |
| Standing (h:min/day) | 1:11 | 1:08-1:15 | 1:16 | 1:12-1:21 | 1:22 | 1:15-1:28 | 7 % | 8 % | 15 % | 0.062 | 0.170 | 0.005 |
| SB (h:min/day) | 9:05 | 8:58-9:12 | 8:59 | 8:51-9:08 | 9:02 | 8:48-9:15 | -1 % | 1 % | -1 % | 0.318 | 0.761 | 0.686 |
| Boys |  |  |  |  |  |  |  |  |  |  |  |  |
| Steps (number/day) | 11197 | 10999-11395 | 11103 | 10910-11395 | 10536 | 10265-10808 | -1 % | -5 % | -6 % | 0.500 | 0.001 | <0.001 |
| Vigorous PA (h:min/day) | 0:19 | 0:18-0:20 | 0:16 | 0:15-0:17 | 0:14 | 0:13-0:15 | -16 % | -13 % | -26 % | <0.001 | 0.019 | <0.001 |
| Moderate PA (h:min/day) | 1:49 | 1:46-1:51 | 1:48 | 1:46-1:50 | 1:41 | 1:39-1:44 | -1 % | -6 % | -7 % | 0.887 | <0.001 | <0.001 |
| MVPA (h:min/day) | 2:08 | 2:05-2:11 | 2:05 | 0:02-2:07 | 1:56 | 1:53-2:00 | -2 % | -7 % | -9 % | 0.072 | <0.001 | <0.001 |
| Light PA (h:min/day) | 3:38 | 3:35-3:40 | 3:50 | 3:48-3:53 | 3:45 | 3:42-3:49 | 6 % | -2 % | 3 % | <0.001 | 0.017 | <0.001 |
| Standing (h:min/day) | 0:45 | 0:44-0:47 | 0:50 | 0:49-0:52 | 0:48 | 0:46-0:50 | 11 % | -4 % | 7 % | <0.001 | 0.041 | 0.035 |
| SB (h:min/day) | 7:59 | 7:55-8:03 | 7:45 | 7:41-7:49 | 8:01 | 7:55-8:07 | -3 % | 3 % | 0 % | <0.001 | <0.001 | 0.679 |
| Girls |  |  |  |  |  |  |  |  |  |  |  |  |
| Steps (number/day) | 9393 | 9261-9525 | 9874 | 9740-10007 | 8943 | 8762-9123 | 5 % | -9 % | -5 % | <0.001 | <0.001 | <0.001 |
| Vigorous PA (h:min/day) | 0:11 | 0:11-0:12 | 0:12 | 0:11-0:12 | 0:09 | 0:08-0:10 | 9 % | -25 % | -18 % | 0.228 | <0.001 | <0.001 |
| Moderate PA (h:min/day) | 1:25 | 1:23-1:26 | 1:30 | 1:28-1:31 | 1:18 | 1.16-1:20 | 6 % | -13 % | -8 % | <0.001 | <0.001 | <0.001 |
| MVPA (h:min/day) | 1:36 | 1:25-1:38 | 1:42 | 1:40-1:43 | 1:28 | 1:25-1:30 | 6 % | -14 % | -8 % | <0.001 | <0.001 | <0.001 |
| Light PA (h:min/day) | 3:59 | 3:57-4:02 | 4:09 | 4:07-4:11 | 4:00 | 3:57 | 4 % | -4 % | 0 % | <0.001 | <0.001 | 0.684 |
| Standing (h:min/day) | 1:04 | 1:03-1:06 | 1:13 | 1:11-1:14 | 1:12 | 1:10-1:14 | 14 % | -1 % | 13 % | <0.001 | 0.763 | <0.001 |
| SB (h:min/day) | 7:45 | 7:42-7:49 | 7:21 | 7:18-7:24 | 7:45 | 7:41-7:50 | -5 % | 5 % | 0 % | <0.001 | <0.001 | 0.926 |
| Weekdays |  |  |  |  |  |  |  |  |  |  |  |  |
| Steps (number/day) | 10925 | 10806-11044 | 11045 | 10928-11161 | 10290 | 10129-10452 | 1 % | -7 % | -6 % | 0.151 | <0.001 | <0.001 |
| Vigorous PA (h:min/day) | 0:16 | 0:15-0:16 | 0:14 | 0:14-0:15 | 0:12 | 0:12-0:13 | -13 % | -14 % | -25 % | <0.001 | <0.001 | <0.001 |
| Moderate PA (h:min/day) | 1:43 | 1:41-1:44 | 1:45 | 1:44-1:46 | 1:35 | 1.34-1:37 | 2 % | -10 % | -8 % | 0.014 | <0.001 | <0.001 |
| MVPA (h:min/day) | 1:59 | 1:58-2:01 | 2:00 | 1:58-2:01 | 1:48 | 1:46-1:50 | 1 % | -10 % | -9 % | 0.568 | <0.001 | <0.001 |
| Light PA (h:min/day) | 3:55 | 3:53-3:56 | 4:02 | 4:01-4:04 | 3:55 | 3:52-3:57 | 3 % | -3 % | 0 % | <0.001 | <0.001 | 0.948 |
| Standing (h:min/day) | 0:58 | 0:56-0:59 | 1:06 | 1:05-1:07 | 1:05 | 1:03-1:07 | 14 % | -2 % | 12 % | <0.001 | 0.296 | <0.001 |
| SB (h:min/day) | 7:49 | 7:46-7:51 | 7:32 | 7:29-7:34 | 7:52 | 7:49-7:56 | -4 % | 4 % | 1 % | <0.001 | <0.001 | 0.116 |
| Weekend days |  |  |  |  |  |  |  |  |  |  |  |  |
| Steps (number/day) | 8466 | 8293-8639 | 9099 | 8931-9267 | 8474 | 8244-8703 | 7 % | -7 % | 0 % | <0.001 | <0.001 | 0.958 |
| Vigorous PA (h:min/day) | 0:12 | 0:11-0:13 | 0:12 | 0:12-0:13 | 0:11 | 0:10-0:12 | 0 % | -8 % | -8 % | 0.464 | 0.002 | 0.014 |
| Moderate PA (h:min/day) | 1:19 | 1:17-1:21 | 1:24 | 1:23-1:26 | 1:17 | 1:14-1:19 | 6 % | -8 % | -3 % | <0.001 | <0.001 | 0.182 |
| MVPA (h:min/day) | 1:31 | 1:29-1:34 | 1:37 | 3:35-1:40 | 1:28 | 1:25-1:31 | 7 % | -9 % | -3 % | <0.001 | <0.001 | 0.058 |
| Light PA (h:min/day) | 3:27 | 3:25-3:30 | 3:49 | 3:47-3:51 | 3:41 | 3:38-3:44 | 11 % | -3 % | 7 % | <0.001 | <0.001 | <0.001 |
| Standing (h:min/day) | 0:47 | 0:45-0.48 | 0:50 | 0:49-0:51 | 0:49 | 0:47-0:50 | 6 % | -2 % | 4 % | <0.001 | 0.178 | 0.081 |
| SB (h:min/day) | 8:04 | 8.00-8:08 | 7:33 | 7.29-7:37 | 7:52 | 7:46-7:57 | -6 % | 4 % | -2 % | <0.001 | <0.001 | <0.001 |
| PA=physical activity, MVPA=moderate-to-vigorous physical activity, CI=confidence interval, SB=sedentary behavior | | | | | | | | |  |  |  |  |
| * Multivariate regression analysis (GLM) adjusted for accelerometer wear time during waking hours, school grade, and sex. | | | | | | | | |  |  |  |  |
